# Positive expression of Midkine predicts early recurrence and poor prognosis of initially resectable combined hepatocellular cholangiocarcinoma

**DOI:** 10.1186/s12885-018-4146-7

**Published:** 2018-02-27

**Authors:** Ming-Chun Ma, Yi-Ju Chen, Tai-Jan Chiu, Jui Lan, Chien-Ting Liu, Yi-Ching Chen, Hsin-Ho Tien, Yen-Yang Chen

**Affiliations:** 1 Division of Hematology-Oncology, Department of Internal Medicine, Kaohsiung Chang Gung Memorial Hospital, 123 Ta-Pei Road, Niaosong District, Kaohsiung, 833 Taiwan; 2 Chang Gung University College of Medicine, Kaohsiung, Taiwan; 3 Department of Anatomic Pathology, E-Da hospital, I-Shou University, Kaohsiung, Taiwan; 4 Kaohsiung Chang Gung Cholangiocarcinoma and Pancreatic Cancer Group, Cancer Center, Kaohsiung Chang Gung Memorial Hospital, Kaohsiung, Taiwan; 5 Institute of Clinical Medical Sciences, Chang Gung University, Kaohsiung, 833 Taiwan; 6 Department of Pathology, Kaohsiung Chang Gung Memorial Hospital, Chang Gung University College of Medicine, Kaohsiung, Taiwan; 7 Department of Nursing, Kaohsiung Chang Gung Memorial Hospital Cancer Center, Kaohsiung Chang Gung Memorial Hospital, Kaohsiung, Taiwan

**Keywords:** Combined hepatocellular cholangiocarcinoma (CHCC-CC), Midkine (MK), Early recurrence, Prognosis

## Abstract

**Background:**

Post-surgical prognosis is usually poor for combined hepatocellular cholangiocarcinoma (CHCC-CC), a rare primary liver cancer. Although midkine (MK) is a prognostic biomarker for several known cancers, it is not known whether it can be used as such in resectable CHCC-CC. This study examined whether MK expression can predict recurrence and survival in patients with resectable CHCC-CC.

**Methods:**

We retrospectively enrolled 52 patients with resectable CHCC-CC who had received curative hepatic resections. MK expression was assessed in post-surgical immunohistochemical studies of specimens in paraffin blocks. Clinical outcomes were analyzed from medical records.

**Results:**

Two-year disease-free and three-year overall survival rates were 42.1% and 44.6%. MK was expressed in 30 patients. Univariate analysis showed patients positively expressing MK had a significantly poorer 2-year disease free and three-year overall survival. Multivariate analysis found positive MK expression independently predicted recurrence.

**Conclusions:**

Positive expression of MK predicts poor prognosis in patients with resectable CHCC-CC.

## Background

Hepatocellular carcinoma (HCC), the most common primary liver malignancy, originates from hepatocytes. Cholangiocarcinoma (CC), the second most common, originates from bile duct epithelial cells. Combined hepatocellular cholangiocarcinoma (CHCC-CC), a rare primary liver cancer consisting of two components, HCC and CC, accounts for 1% to 14.3% of all primary malignant liver tumors [1–9]. To date, complete tumor resection is its only possible cure. It is difficult to precisely diagnose this disease preoperatively. Although CHCC-CC is usually diagnosed post-operatively based on pathological findings, immunohistochemical stains have also been used to further confirm the presence of both components [6, 8, 10, 11].

The clinicopathological characteristics and prognosis of patients with CHCC-CC after surgery have not been reported in detail because the incidence of this disease is very low and studies on it are scarce. While these studies report conflicting outcomes, most conclude that CHCC-CC has a worse prognosis than either HCC or CC alone [12]. Only a few papers discuss the possible molecular markers that could be used to predict outcome in CHCC-CC [13, 14]. Because the molecules studied were not found to efficaciously predict likelihood of post-surgical CHCC-CC recurrence, there remains a need to identify biomarkers that can help assess its treatment response as well as predict recurrence and prognosis.

Midkine (MK) is a heparin-binding growth factor weakly expressed or undetectable in normal adult tissue but strongly expressed during embryogenesis [15]. It is expressed at abnormally high levels in several human cancers, including esophagus, gall bladder, pancreas, colorectal, breast, salivary gland and lung carcinomas [16–18]. It has been found to exacerbate disease by promoting many tumor specific functions, including cell growth, tumor cell survival, cell migrations, and carcinogenesis [19–21].

Some studies have investigated the possibility of using MK as a biomarker to predict prognosis and assess response to treatment in oral squamous cell carcinoma and report that its positive expression predicts poor prognosis in patients with various malignant tumors, including head and neck squamous cell carcinoma [22], esophageal squamous cell carcinoma [23] and non-small cell lung cancer [24]. Although MK is known to be overexpressed in hepatocellular carcinoma [25] and cholangiocarcinoma [26], little is known about its significance in CHCC-CC. Therefore, this study investigated the relationship between its expression and the pathogenesis of CHCC-CC as well as the disease’s clinicopathology and survival.

## Methods

### Patients

In this retrospective study, we collected medical records of 52 patients with primary CHCC-CC treated with surgical resection between January 2000 and December 2013 at Kaohsiung Chang Gung Memorial Hospital in Kaohsiung, an industrial city located in southern Taiwan. We also performed immunohistochemical studies of tissue samples collected during surgery. Following Allen and Lisa classification [2], we included only patients with CHCC-CC classified as type C (intimate intermingling of hepatocellular and glandular elements) and type B (contiguous but independent masses of HCC and CC). We excluded patients with type A (separate masses constituting either HCC or CC). CHCC-CC was diagnosed pathologically based on microscope studies of thin-section specimens stained with hematoxylin and eosin. The immunoreactivity of each tumor was confirmed: hepatocyte paraffin 1 (Hep1) antibody and CK-7 (cytokeratin-7) in CHCC-CC.

We retrospectively reviewed the medical records of all the patients with CHCC-CC to obtain medical histories of the present illness. Pre-surgery laboratory data, including tumor markers, serum viral markers, and radiologic evaluations, were recorded. Serum AFP, carbohydrate antigen 19-9, and carcinoembryonic antigen levels before and after tumor resection were recorded, when data were available. If patients had positive findings for HBsAg and anti-HCV Ab for more than 6 months, they were assumed to have chronic hepatitis B and C infections. Tumor staging was performed in accordance with the American Joint Committee on Cancer staging system sixth edition [27]. Cumulative recurrence rate, median disease-free survival, and median overall survival were calculated. Information was also collected on suspected prognostic factors, including AFP, CEA, and CA-199 levels, seropositivity for HBsAg or anti-HCV Ab, sex, and tumor stage.

Informed consent was obtained in written form from all study participants and the protocol for this study was approved by the Institutional Review Boards of Chang-Gung Medical Center (Taiwan) (IRB 103-7412B).

#### Immunohistochemical study

A pathologist with expertise in hepatic tumors reviewed macroscopic and microscopic pathological findings and concluded that both HCC cells and ICC cells coexisted in liver tumors and that ICC cells made up more than 10 % of the cells within the tumor. Hematoxylin and Eosin (H&E) as well as immunohistochemical staining were performed. HCC cells were confirmed based on immunohistological stains of hepatocyte paraffin 1 (Hep1) and ICC cells confirmed based on cytokeratin 7 (CK7).

Immunohistochemical studies were performed to measure protein levels of MK in paraffin sections of samples obtained from all 52 patients. Samples were fixed with 10% buffered formalin embedded in paraffin, deparaffinized with xylene and rehydrated in a series of ethanol washes (100d%, 90%, 80% and 70%), and subsequently washed with phosphate-buffered saline (PBS) followed by treatment with 3% H2O2 for 30 min to block endogenous peroxidase activity. Sections were then microwaved in 10 mM citrate buffer (pH 6.0) to unmask the epitopes, incubated with MK monoclonal antibody (Abcam Plc, Cambridge, UK) for 1 h, and washed with PBS. The reaction was visualized using horseradish peroxidase/Fab polymer conjugate (PicTure™-Plus kit Zymed, South San Francisco, CA) and diaminobenzidine. An antibody assay without the primary antibody was used as a negative control.

MK immunostaining was evaluated independently by two pathologists blinded to the subjects’ clinical information. Each specimen was assigned a score of 1 to 4 based on the percentage of positive cells within a field of cells (100 x magnification): one for < 5% of the cells, two for 6–35% of the cells, three for 36–70% of the cells, and 4 for > 71% of the cells. Each specimen also received another score of 1 to 4 based on intensity of staining: one for negative staining, two for weak staining, three for moderate staining and four for strong staining. MK expression score was then calculated by multiplying the percentile and intensity scores. A score of ≥4 for MK protein expression levels indicated the tumor was positive [16].

#### Immunofluorescent staining

Immunofluorescent staining was performed on paraffinized tumor samples obtained from the CHCC-CC patients to confirm the expression of CK7 (Abcam) and Hep1(Abcam). After deparaffinization, the sections were blocked with 5% normal goat serum/PBS at room temperature for 1 h. They were then incubated with primary antibody (CK7; 1:100, Hec-1; 1:100) at 4 °C overnight. Next they were each washed three times with PBS for 5 min. After incubation with secondary antibodies (1:1000) at room temperature for 1 h, they were each washed three times with PBS for 5 min. They were observed under a confocal microscope.

#### Statistical analysis

Patients’ demographic information and clinical characteristics were analyzed by MK expression percentile and compared using either Pearson’s chi-square test or Fisher’s exact test. Overall and disease-free survival were calculated and compared by Log-rank test. (if cell numbers were less than five). Overall and disease-free survival were calculated and compared by Log-rank test. Overall survival was calculated starting from the date that adjuvant treatment was initiated to date of the patient’s death or most recent follow-up. Disease-free survival was calculated starting from the date of operation to the date of the first indication of disease progression, disease relapse or death due to any cause. Binary logistic regression was used to analyze the association between positive MK expression and patients’ demographic and clinical characteristics. Results were reported as odd ratio (OR) with 95% confidence interval (95% CI). Step-wise Cox-regression analysis was performed to evaluate whether there was an association between patients’ demographic and clinical characteristics and overall and disease-free survival. If any of these variables were found to be significantly associated with these two outcomes (*p* < 0.05) in univariable Cox-regression, we further analyzed their association using multivariable Cox-regression. Results were expressed as hazard ratio (HR) with 95% confidence interval of (95% CI) and corresponding *p* values. All statistical operations were performed using SPSS 13.0 statistics software (SPSS Inc., Chicago, IL).

## Results

### Patient characteristics

Patients (37 male; 71.2%) had a mean age of 58 years±11.35 (range 32-83 years). Thirty-five patients (67.3%) were found positive for HbsAg, 16 (30.8%) positive for HCV Ab alone, and 8 (15.4%) for both. Seventeen patients (58.6%) had LC, 26 (50.0%) Child A, 7 (13.5%) Child B, and 5 (9.6%) Child C. Fourteen patients (26.9%) had diabetes mellitus (DM) and 5 (9.6%) had gall stones. Preoperative AFP levels were above normal (> 15 ng/ml) in 29 patients (55.8%), CEA levels were above normal (> 5 ng/ml) in 7 patients (13.5%), and CA 19-9 levels were above normal (> 37 U/ml) in 16 (30.8%). Based on the American Joint Committee on Cancer staging system, at the time of resection, 16 patients (30.8%) had stage I tumors, 24 (46.2%) stage II, 8 (15.4%) stage III and 4 (7.7%) stage IVA. No patient had stage IVB [(any T any N M1): distant metastasis (M1)] disease.

### Correlation between MK and clinicopathological characteristics in CHCC-CC patients

**Fig. 1:**
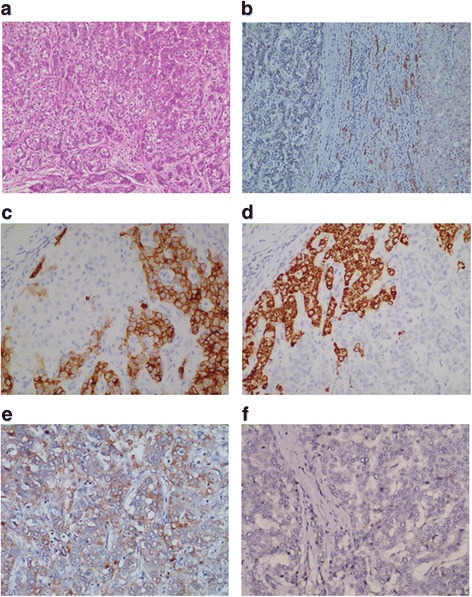
Pathological features and MK expression in combined hepatocellular cholangiocarcinoma (CHCC-CC). Microscopically, the tumor was diagnosed as CHCC-CC with HE staining (**a**). Adjacent non-cancerous tissue showed no MK expression (**b**). Because the HCC component was positive with hep1 but negative with CK7 (**c**). Conversely, ICC component was positive with CK7 but negative with hep1 (**d**). Positive MK expression in some CHCC-CC tissues (**e**). Negative MK expression in some CHCC-CC tissues (**f**)

**Fig. 2:**
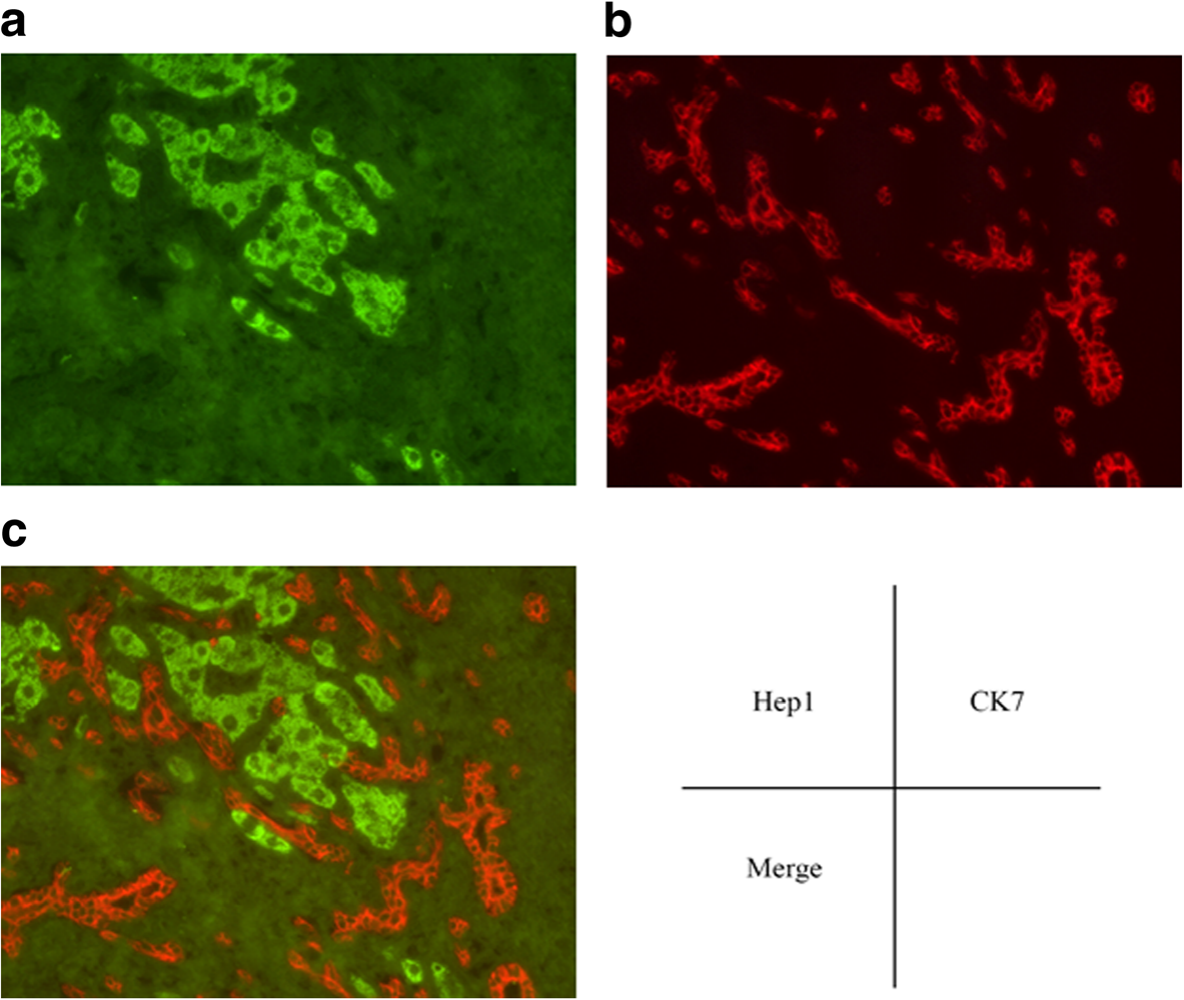
Two-color immunofluorescent staining for CK7 and Hep1 in the CHCC-CC patient samples. Hep1 (**a**), CK7 (**b**) and two color immunofluorescent stain merge (**c**)

Immunohistochemical staining was performed on 52 paraffin-embedded CHCC-CC tissue samples (Figs. 1a-1f). Two-color immunofluorescent staining for CK7 and He1 confirmed both HCC and ICC cell markers were expressed in the serial sections of CHCC-CC (Fig. 2a-c). As can be seen in Table 1, 30 of the 52 patients (57.7%) positively expressed MK. There were no significant correlations between that expression and gender (*p* = 0.942), age at diagnosis (*p* = 0.516), hepatitis B (*p* = 0.679), hepatitis C (*p* = 0.870), liver cirrhosis (*p* = 0.753), diabetes mellitus (*p* = 0.753), gall stone (*p* = 0.149), histological grade (=0.753), lymph-vascular invasion (*p* = 0.253), peri-neural invasion (*p* = 0.226), surgical margin (*p* = 0.679), lymph nodes metastases (*p* = 1.000) or AJCC tumor stage (*p* = 0.094). There was, however, a significant association between high expression of MK protein and clinically advanced T stage (T3/T4 vs T1/T2; *p* = 0.007) (Table 1).

**Table 1:** Relationships between midkine expression and clinicopathological factors

Midkine	Negative expression	Positive expression	*P*
Age			
< 60	15 (53.6%)	13 (46.4%)	0.516
≥ 60	15 (62.5%)	9 (37.5%)	
Gender			
Male	22 (59.5%)	15 (40.5%)	0.924
Female	8 (53.3%)	7 (46.7%)	
Hepatitis B			
Negative	11 (64.7%)	6 (35.3%)	0.679
Positive	19 (54.3%)	16 (45.7%)	
Hepatitis C			
Negative	20 (55.6%)	16 (44.4%)	0.870
Positive	10 (62.5%)	6 (37.5%)	
Liver cirrhosis			
No cirrhosis	9 (64.3%)	5 (35.7%)	0.753
Cirrhosis	21 (55.3%)	17 (44.7%)	
DM			
Negative	21 (55.3%)	17 (44.7%)	0.753
Positive	9 (64.3%)	5 (35.7%)	
Gall stone			
Negative	29 (61.7%)	18 (38.3%)	0.149
Positive	1 (20.0%)	4 (80.0%)	
CEA			
≤ 5	27 (60.0%)	18 (40.0%)	0.438
> 5	3 (42.9%)	4 (57.1%)	
CA-199			
≤ 35	20 (55.6%)	16 (44.4%)	0.870
> 35	10 (62.5%)	6 (37.5%)	
AFP			
≤ 15	12 (52.2%)	11 (47.8%)	0.473
> 15	18 (62.1%)	11 (37.9%)	
Histology grade			
Well and moderately	21 (55.3%)	17 (44.7%)	0.753
Poorly	9 (64.3%)	5 (35.7%)	
LVI			
Negative	21 (63.3%)	12 (36.4%)	0.253
Positive	9 (47.4%)	10 (52.6%)	
PNI			
Negative	25 (54.3%)	21 (45.7%)	0.226
Positive	5 (83.3%)	1 (16.7%)	
Surgical margin			
< 10 mm	19 (54.3%)	16 (45.7%)	0.679
≥ 10 mm	11 (64.7%)	6 (35.3%)	
T stage			
T1-T2	28 (66.7%)	14 (33.3%)	0.007*
T3-T4	2 (20.0%)	8 (80.0%)	
N stage			
Negative	28 (57.1%)	21 (42.9%)	1.000
Positive	2 (66.7%)	1 (33.3%)	
AJCC staging			
I-II	26 (65.0%)	14 (35.0%)	0.094
III-IV	4 (33.3%)	8 (66.7%)	

*DM* diabetes Mellitus, *LVI* lymph-vascular invasion, *PNI* peri-neural invasion

### Survival analysis

**Fig. 3:**
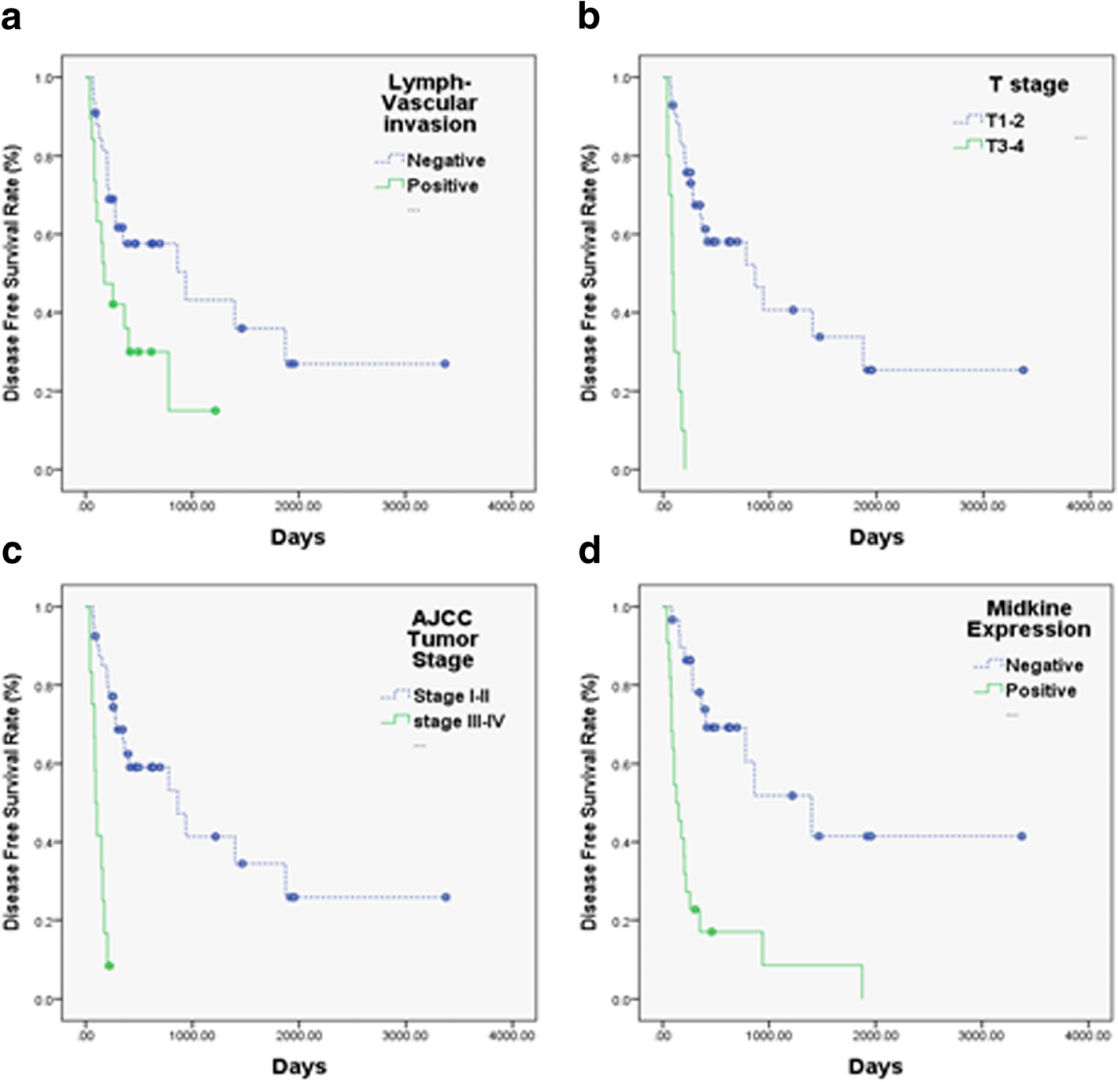
Kaplan-Meier estimates of the probability of disease free survival (DFS). Positive Lymph-vascular invasion (LVI) (**a**), T-stage III/IV (**b**), AJCC tumor stage III/IV (**c**) and Positive MK expression (**d**) were associated with poor DFS

**Fig. 4:**
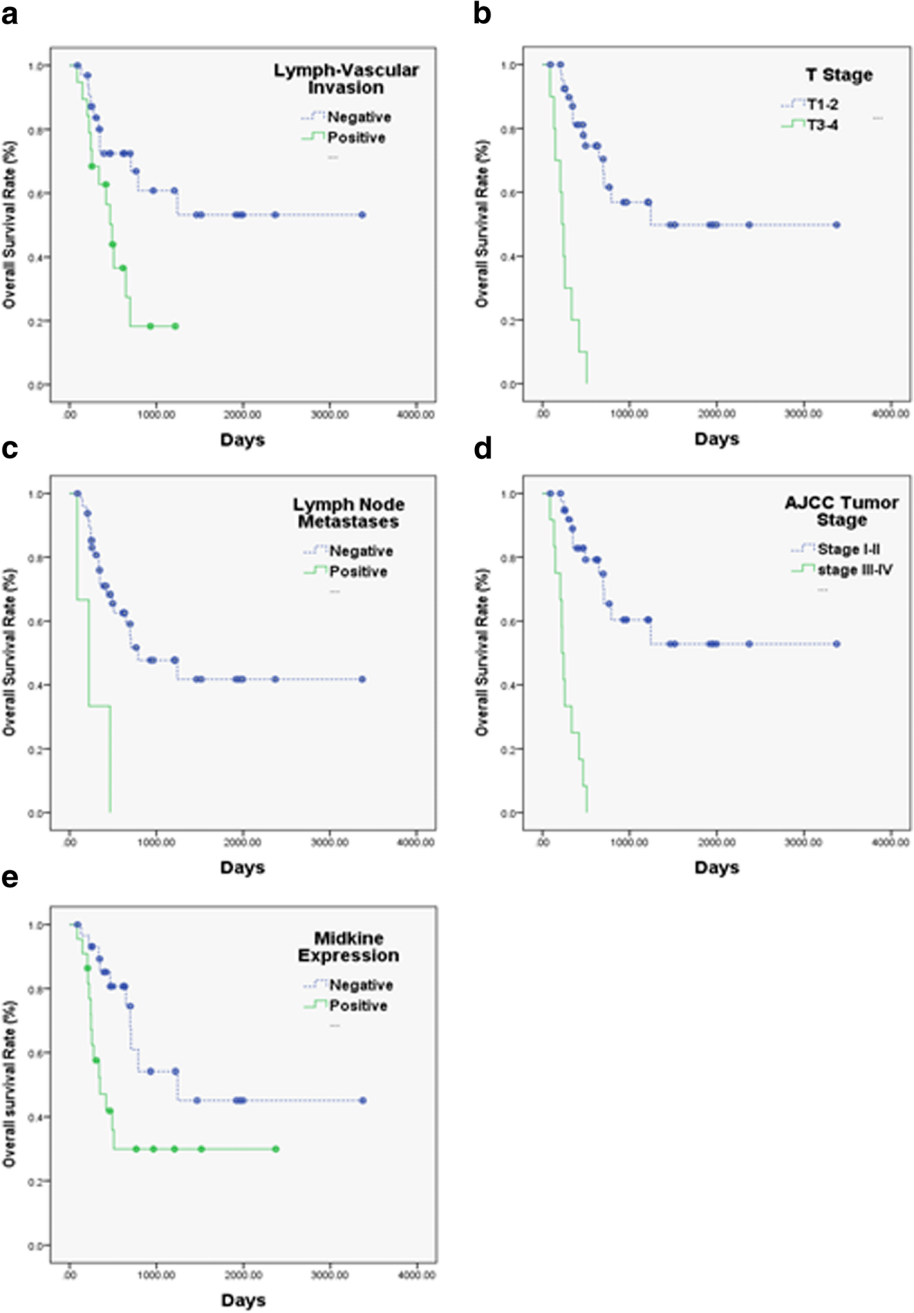
Kaplan-Meier estimates of the probability of overall survival (OS). Positive lymph-vascular invasion (LVI) (**a**), T stage III/IV (**b**), Node positive (**c**), AJCC tumor staging III/VI (**d**) and positive MK expression (**e**) had poorer OS

Median follow-up was 688 days (87-3374 days). Twenty-four patients died. Median disease free survival was 513 days, two-year disease free survival was 42.1%, and three-year overall survival 44.6%. As shown in Table 2, disease free survival was significantly lower in patients with positive lymph-vascular invasion (*p* = 0.022, Fig. 3a), T stage III/IV (*p* < 0.001, Fig. 3b), AJCC tumor staging III/IV (*p* < 0.001, Fig. 3c) and MK expression (*p* < 0.001, Fig. 3d). Overall survival was also significantly lower in patients with lymph-vascular invasion (*p* = 0.009, Fig. 4a). It was also significantly lower in patients with positive lymph-vascular invasion (LVI) (*p* = 0.009, Fig. 4a), T3/4 stage (*p* < 0.001, Fig. 4b), positive N stage (*p* = 0.001, Fig. 4c), AJCC tumor staging III/V (*p* = < 0.001, Fig. 4d) and positive MK expression (*p* = 0.012, Fig. 4e).

**Table 2:** Correlation between the clinicopathological features and 2-year progression-free survival in combined hepatocellular cholangiocarcinoma

Variables	No. of patients	Cumulative 2- year progression-free survival rate	P	HR (95% CI)	*P*
Age					
< 60	28	38.5%	0.941		
≧60	24	35.6%			
Gender					
Male	37	35.8%	0.097		
Female	15	59.3%			
Hepatitis B					
Negative	17	39.7%	0.632		
Positive	35	44.9%			
Hepatitis C					
Negative	36	42.5%	0.747		
Positive	16	41.5%			
Liver cirrhosis					
No cirrhosis	14	55.1%	0.479		
Cirrhosis	38	39.2%			
DM					
Negative	38	44.7%	0.575		
Positive	14	37.5%			
Gall stone					
Negative	47	45.2%	0.178		
Positive	5	20.0%			
CEA					
≤ 5	45	44.3%	0.311		
> 5	7	34.3%			
CA-199					
≤ 35	36	43.7%	0.535		
> 35	16	19.3%			
AFP					
≤ 15	23	26.6%	0.978		
> 15	29	43.5%			
Histology grade					
Well or Moderate	38	44.9%	0.499		
Poorly	14	40.8%			
LVI					
Negative	33	50.4%	0.022*		
Positive	19	15.0%			
PNI					
Negative	46	44.1%	0.377		
Positive	6	33.3%			
Surgical margin					
<10 mm	35	35.4%	0.746		
≥ 10 mm	17	41.6%			
T stage					
TI-II	42	52.3%	< 0.001*	8.004 (2.869-22.336)	< 0.001*
TIII-IV	10	0%			
N stage					
Negative	49	43.3%	0.223	4.701 (0.952-23.203)	0.057
Positive	3	33.3%			
AJCC staging					
I-II	40	53.2%	< 0.001*		
III-IV	12	8.3%			
Midkine expression					
Negative	30	60.5%	< 0.001*	4.238 (1.900-9.449)	< 0.001*
Positive	22	8.5%			

*CI* confidence interval, *HR* hazard ratio

Multivariable analysis revealed T stage and positive MK expression to be independent prognostic factors for disease-free survival after resection (HR = 8.004 and HR = 2.240; both *p* < 0.001) (Table 2) and AJCC tumor stage was found to be an independent prognostic factor for overall survival (HR = 12.784, *p* < 0.001) (Table 3).

**Table 3:** Correlation between the clinicopathological features and 3-year overall survival in combined hepatocellular cholangiocarcinoma

Variables	No. of patients	Cumulative 3- year Overall survival rate	P	HR (95% CI)	*P*
Age					
< 60	28	46.1%	0.962		
≧60	24	41.9%			
Gender					
Male	37	41.7%	0.531		
Female	15	58.8%			
Hepatitis B					
Negative	17	15.1%	0.208		
Positive	35	58.8%			
Hepatitis C					
Negative	36	49.4%	0.548		
Positive	16	29.8%			
Liver cirrhosis					
No cirrhosis	14	49.9%	0.649		
Cirrhosis	38	45.7%			
DM					
Negative	38	44.5%	0.816		
Positive	14	44.9%			
Gall stone					
Negative	47	49.7%	0.109		
Positive	5	0%			
CEA					
≤ 5	45	48.6%	0.052		
> 5	7	0%			
CA-199					
≤ 35	36	52.6%	0.069		
> 35	16	24.6%			
AFP					
≤ 15	23	42.7%	0.825		
> 15	29	45.6%			
Histology grade					
Well or Moderate	38	49.3%	0.328		
Poorly	14	22.4%			
LVI					
Negative	33	60.8%	0.009*		
Positive	19	18.3%			
PNI					
Negative	46	48.2%	0.060		
Positive	6	0%			
Surgical margin					
< 10 mm	35	41.2%	0.552		
≥ 10 mm	17	61.6%			
T stage					
TI-II	42	56.9%	< 0.001*		
TIII-IV	10	0%			
N stage					
Negative	49	47.7%	0.001*		
Positive	3	0%			
AJCC staging					
I-II	40	60.4%	< 0.001*	12.784 (4.822-33.481)	< 0.001*
III-IV	12	0%			
Midkine expression					
Negative	30	54.2%	0.012*		
Positive	22	29.9%			

*CI* confidence interval, *HR* hazard ratio

## Discussion

The incidence of CHCC-CC, a rare tumor, varies among different studies. The actual incidence may be higher than usually reported for two reasons. First, because most patients have either no symptoms or non-specific symptoms, clinical presentation does not generally contribute to diagnosis. Second, patients with CHCC-CC frequently present more advanced disease stages than those with typical HCC, and so a high percentage of patients with CHCC-CC do not receive surgical resection [28]. It is very difficult to make accurate diagnosis of CHCC-CC before surgery, because CT or MRI scans often do not show typical patterns of contrast uptake or washout. CHCC-CC may have CT features of both HCC and CC when a hepatic tumor contains an area of hyper-enhancement in the early phase and an area of delayed enhancement in the late phase on dynamics CT [29]. It may also have features of both when a hypovascular liver tumor is associated with significant elevation of a-fetoprotein levels and multiple regional lymph nodes metastases [30], or when a hepatic tumor has hypervascular expression and elevation of serum CEA and carbohydrate antigen 199. Although histological studies may be able to identify a dominant tumor type, they usually fail to detect the presence of both CHCC and CC. Therefore, precise diagnosis of CHCC-CC before surgery remains a challenge.

Some studies report CHCC-CC to have a worse prognosis than either HCC or CC alone [3, 9]. Similarly, our 52 CHCC-CC patients, who had received intensive curative tumor resection, had a two-year disease free survival of 42.1% (median 17.3 months) and a three-year overall survival of 44.6% (median 22.9 months). Because most of the CHCC-CC patients in this study were male patients with a high prevalence of HBV, chronic HBV infection may be a major cause for both HCC and CHCC-CC in Taiwan. The factors predicting survival also vary among different studies, possibly due to the limited number of patients [12]. Univariate analyses performed by prior studies found overall survival to be significantly related to microscopic vascular invasion, bilobar tumors and tumors > 6 cm [9], vascular invasion and satellite lesions [3], and lymph node metastases. Univariate analysis of our patients’ data found LVI, T stage III-IV, AJCC tumor stage III-IV and positive midkine expression to be associated with poorer disease free and overall survival. Our multivariate analysis revealed AJCC tumor stage III-IV to be the most important predictor of both survival rates. These findings are consistent with previous CHCC-CC studies [3, 31, 32].

In this study, positive expression of MK in tumors was associated with poor prognosis and reduced survival in CHCC-CC patients, suggesting that MK could potentially be used as an independent post-surgical prognostic biomarker for CHCC-CC. MK expression predicted poor prognosis in disease free survival in both our univariate and multivariate analyses. Yoon et al. [33] attributed poor overall survival in this population to shorter survival after recurrence. Although our multivariate analysis did not find a significant association between MK expression and overall survival, our univariate analysis did, though the small number of cases may have affected this result.

MK, a heparin-binding growth factor, plays a central role in chemotaxis, angiogenesis, and the inhibition of apoptosis. The expression of MK is elevated in various tumors [34], and has been described as a potential prognostic marker in several malignancies, including esophageal cancer [35], endometrial carcinoma [36] and gastric cancer [37]. One study found breast cancer patients to have higher plasma levels of midkine than their healthy controls [38]. Another study also found a correlation between MK protein expression and malignant status and prognosis of breast cancer patients [39]. Zhu et al. found serum MK levels to be clearly increased in hepatocellular cancer patients and suggested they could be used to diagnose hepatocellular cancer with a high sensitivity. Moreover, serum MK levels, which were reported by one study to be markedly decreased in hepatocellular cancer patients after curative resection, were found by the same study to re-increased when tumors recurred [40]. Keto et al. also reported MK to be increased at messenger RNA and protein levels in patients with intrahepatic cholangiocarcinoma, although they stated that the ultimate biological significance and their possible relationship to tumor behavior had not been established. To the best of our knowledge, the current study is the first to report the results of MK immunohistochemical analysis of CHCC-CC tissue.

Cancer stem cell markers have been correlated with poor prognosis in primary liver malignancy and their presence has been associated with carcinogenesis, vascular invasion, and metastasis in this disease [41–43]. Therefore, the 2010 WHO classification divides CHCC-CC into two subtypes: the classical type and subtypes with stem cell features [44]. Classical type CHCC-CC includes HCC areas, CC areas and transitional zones, which comprise tumor cells with stem cell features. MK is known to have the ability to induce epithelial mesenchymal transition (EMT) in some types of cancer cells, the differentiation of lost polarity in epithelial cells and cell adhesion to contractile and motile mesenchymal cells [45]. In addition to angiogenesis, Takenaka et al. found that the growth of mouse embryonic stem cells could be induced while MK inhibits apoptosis through the PI3K/Akt signaling pathway [46]. Zhao et al. reported that mesenchymal stem cells overexpressing MK transplantation stimulate vasculogenesis effectively by increasing pro-angiogenesis factors (VEGF, TGF-β). These findings suggest that MK might be involved in the pathogenesis of liver cancers with stemness. Our study showed the patients with CHCC-CC who had a positive expression of MK to be at much higher risk of early recurrence and poor survival. However, currently MK is still not a suitable as therapeutic target and drugs need to be developed for this in the future.

This study has several limitations. First, it was based on a retrospective analysis of data collected from only 52 patients accumulated over a short period. Second, only three enrolled CHCC-CC patients had regional lymph node metastases. Preoperative diagnosis for patients with CHCC-CC is very difficult because there is no typical pattern of contrast uptake or washout in dynamic CT or MRI scans. In our series, most patients were diagnosed as having HCC before surgery. Patients who are found to have image study evidence of lymph node involvement before treatment often receive systemic treatment instead of hepatectomies, since HCC with lymph node metastases post curative resection has been reported to have poor disease free and overall survival [47]. Third, therapies used to treat recurrent CHCC-CC may affect overall survival. Some studies showed the recurrence rates following resections of these tumors were high and these recurrent tumors were commonly detected in the remnant liver [12]. Salvage treatment can include surgery, trans-arterial embolization, percutaneous ethanol injection, and radiofrequency ablation.

## Conclusions

In conclusion, the findings of the present study suggest that heparin-binding growth factor MK contributes to the clinical outcome in patients with resectable CHCC-CC. It is significantly elevated in patients with advanced T-stage CHCC-CC. A high level of MK protein independently predicts a poor prognosis for patients receiving surgery for this disease.
